# Accuracy of the Standard and Distal-to-Proximal Sequence of the Upper Limb Neurodynamic Test 1 for the Diagnosis of Carpal Tunnel Syndrome: The Role of Side-to-Side Comparisons

**DOI:** 10.3390/jcm13237122

**Published:** 2024-11-25

**Authors:** Gianluca Ciuffreda, Elena Bueno-Gracia, María Irache Argüello-Espinosa, Michael Shacklock, Sonia Navarrete-Navarro, Inés Vicente-Garza, Diego Rodríguez-Mena, Elena Estébanez-de-Miguel

**Affiliations:** 1 Department of Human Anatomy and Histology, Faculty of Health Sciences, University of Zaragoza, Calle Domingo Miral S/N, 50009 Zaragoza, Spain; 2 PhysiUZerapy: Health Sciences Research Group, University of Zaragoza, Calle Domingo Miral S/N, 50009 Zaragoza, Spain; elesteba@unizar.es; 3 Department of Physiatry and Nursing, Faculty of Health Sciences, University of Zaragoza, Calle Domingo Miral S/N, 50009 Zaragoza, Spain; 4 Neurology Department, University Clinical Hospital Lozano Blesa, C. San Juan Bosco 15, 50009 Zaragoza, Spain; 5 Neurodynamic Solutions, Adelaide 5034, Australia; shacklock@yahoo.com; 6 Neurophysiology Department, University Clinical Hospital Lozano Blesa, C. San Juan Bosco 15, 50009 Zaragoza, Spain; snavarreten@salud.aragon.es (S.N.-N.); inesvicente3@gmail.com (I.V.-G.); drodriguezm@salud.aragon.es (D.R.-M.)

**Keywords:** neurodynamic sequencing, carpal tunnel syndrome, median nerve, diagnosis

## Abstract

**Background/Objective**: This study aimed to evaluate the diagnostic accuracy of two upper limb neurodynamic test 1 (ULNT1) sequences for detecting carpal tunnel syndrome (CTS) in patients with unilateral symptoms. The standard sequence (ULNT1-STD) and a distal-to-proximal sequence (ULNT1-DIST) were investigated. A local-initiated sequence may facilitate symptoms reproduction in CTS, and comparing the affected side with the unaffected side could improve the detection of altered median nerve mechanosensitivity when symptoms are not directly reproduced. **Methods**: A total of 134 consecutive patients with clinically suspected unilateral CTS were recruited. Nerve conduction studies were used as a reference test. **Results**: When considering only symptom reproduction as the criterion for a positive test, ULNT1-STD showed a sensitivity of 0.398 and a specificity of 0.780 (positive likelihood ratio [+LR]: 1.81; negative likelihood ratio [−LR]: 0.77); whereas ULNT1-DIST demonstrated a sensitivity of 0.548 with a specificity of 0.732 (+LR: 2.04; −LR: 0.62). When a positive test was defined by symptom reproduction or inter-limb asymmetry (in range of motion or sensory response), ULNT1-STD showed an improved sensitivity of 0.613 but a reduced specificity of 0.537 (+LR: 1.32; −LR: 0.72). In comparison, ULNT1-DIST increased its sensitivity to 0.871 with a specificity of 0.683 (+LR: 2.75; −LR: 0.19). **Conclusions**: ULNT1-DIST offers better diagnostic accuracy for CTS compared to the ULNT1-STD sequence, especially when interlimb asymmetries in range of motion or sensory response are considered. However, side-to-side comparisons have reduced utility in cases with bilateral symptoms, limiting their application in clinical practice.

## 1. Introduction

Carpal tunnel syndrome (CTS) is currently considered the most common entrapment neuropathy, caused by the compression of the median nerve within the carpal tunnel [1]. The prevalence of CTS in the general population ranges from 2.7% to 4.9% [2] and has shown a progressive increase over time [3].

Diagnosis is commonly confirmed with nerve conduction studies (NCSs) [1,4,5], although other techniques, such as high-resolution ultrasound, diffusion tensor imaging, or high-field magnetic resonance imaging, have been proposed in recent years [6,7,8,9]. Even with these advancements, clinical evaluation remains an important initial step in the diagnostic process of CTS [1,4,10,11]. Clinical evaluation includes collecting personal information, observing deformities, assessing range of motion, strength, and sensibility, administering questionnaires, and performing various provocative tests [1,4,10,11].

Neurodynamic tests (NDTs) have gained relevance as provocative tests for evaluating neuropathic pain [12,13,14,15,16,17,18]. Neurodynamic tests apply a series of movements designed to load the nervous system and produce a response that is classified as normal or pathological based on its characteristics [12,18,19]. Clinically, NDTs are used to assess various conditions affecting the peripheral nervous system [12,13,15,16,17,18,20,21,22]. When the nervous system is affected, an increased mechanosensitivity is often present, which can be detected using NDTs [12,18,19,23,24,25,26].

Specifically, the upper limb neurodynamic test 1 (ULNT1) has been studied as a clinical test for diagnosing CTS [20,27,28,29,30]. Different studies report a positive likelihood ratio (+LR) ranging from 0.86 to 3.67 and a negative likelihood ratio (−LR) from 0.50 to 1.90 [11,14,21]. A recent meta-analysis found an overall sensitivity of 75% and a specificity of 13% for ULNT1 in diagnosing CTS [10]. Due to the heterogeneity of the results and the relatively low diagnostic values reported, ULNT1 is currently not among the most recommended provocative tests for CTS diagnosis [10,11,21].

It is important to note that the interpretation of an NDT plays a key role in determining whether the test results are classified as normal or pathological. In various studies, ULNT1 has been considered positive for CTS when at least one of the following criteria was met: the reproduction of the patient’s symptoms, an interlimb range of motion (ROM) difference exceeding 10°, or any symptom change with cervical side flexion [27,29,30]. Other studies focused on response location, and defined a positive ULNT1 if any symptoms were elicited in the hand or first three fingers [20,28,29]. Some researchers have suggested that this excessively liberal definition of a positive NDT in certain studies may have influenced the reported diagnostic values for CTS [21].

In line with this, some aspects regarding NDT interpretation must be considered.

First, the joint movements required during an NDT may affect non-neural tissues [12,18,19]. Therefore, to distinguish whether the symptoms produced by the NDT are originated by neural or musculoskeletal tissues, structural differentiation (SD) has been recommended [12,18,19,20,21,31,32,33,34,35,36,37,38,39,40]. This technique involves moving a body part far from the area of the elicited response to increase or decrease mechanical load on the nervous system [12,18,19,20,21,31,32,33,34,35,36,37,38,39,40]. However, only one study considered the results of SD to determine neural involvement when ULNT1 reproduced patients’ clinical symptoms [20].

Moreover, with NDTs, healthy subjects usually experience normal neural-related responses (i.e., with positive SD) [18,38,41,42], which, in the case of ULNT1, are commonly located in the hand and fingers [18,38,42]. Hence, considering ULNT1 positive for CTS only because it shows positive SD regardless the reproduction of patient’s clinical symptoms, or if a response appears in the hand or first three fingers, may result in a high rate of false positives, as these criteria could easily include healthy individuals [18,38,42].

Furthermore, at the end of an NDT, a reduced ROM in the affected side compared to the unaffected side may indicate increased nerve mechanosensitivity, leading to less tolerance to tension [18,26,43,44,45,46,47]. According to this, some of the validity studies mentioned earlier have considered ULNT1 positive for CTS if asymmetries in ROM were found between limbs [27,29,30]. However, since these studies involved participants with bilateral symptoms, side-to-side differences may have been compromised, which could have influenced the diagnostic values of ULNT1 [27,29,30].

To date, no diagnostic accuracy studies have investigated ULNT1 by comparing interlimb responses and ROM in a sample with unilateral suspicion of CTS. Using the asymptomatic side as a reference could facilitate the detection of signs of increased mechanosensitivity in the affected nerve, thereby improving the clinical utility of the test for CTS detection.

In addition to side-to-side comparisons, some authors suggest using specific movement sequences during an NDT to increase mechanical stress on particular nerve segments (i.e., neurodynamic sequencing) [18,48,49,50,51,52]. Since nerves do not behave uniformly along their course [18,48,50,52,53,54,55,56], the load on a specific nerve segment may be greater if the nearest joints are moved first [49,52,54,56,57]. Based on this, using an NDT with a local started sequence may increase the test accuracy to detect disorder in that area [12,18,49]. For example, starting ULNT1 with cervical contralateral lateral flexion might enhance its accuracy for detecting cervical radiculopathy, whereas starting with wrist dorsiflexion might increase its accuracy for diagnosing CTS. In this line, Bueno-Gracia et al. (2024) [51] found a high specificity in CTS diagnosis for ULNT1 using neurodynamic sequencing. However, a direct comparison with the standard sequence was not performed [51]. Consequently, it remains unclear in clinical practice whether performing ULNT1 with a locally initiated movement sequence would enhance its diagnostic capabilities for specific conditions respect to the standard sequence.

Therefore, this study aims to investigate the diagnostic accuracy of ULNT1 using the standard sequence (ULNT1-STD) [18] and a distal-to-proximal sequence (ULNT1-DIST) for detecting CTS in a sample with unilateral symptoms. Using a local-initiated sequence could facilitate symptom reproduction in CTS, and comparing the affected side to the unaffected side may improve the detection of altered median nerve mechanosensitivity when the test does not directly reproduce the patient’s symptoms.

## 2. Materials and Methods

### 2.1. Study Design

A prospective diagnostic accuracy study was conducted between June 2023 and September 2024 following the STARD 2015 guidelines [58]. ULNT1-STD and ULNT1-DIST represented the index tests. Nerve conduction studies were used as the reference standard for diagnosing CTS [20,27,28,29,30]. The study was approved by the Aragon Institutional Ethical Committee (CEICA, reference: C.I. PI23/099), and all procedures were conducted according to the Declaration of Helsinki (last modified in 2013) [59]. All participants provided written informed consent prior to participating.

### 2.2. Participants

Consecutive patients with clinical suspicion of CTS referred by their general practitioner or medical specialist to the Clinical Neurophysiology Department of Lozano Blesa University Hospital in Zaragoza for NCS were eligible to participate.

Patients were included if they were aged ≥ 18 years, presented unilateral symptoms, and had adequate comprehension and communication abilities. Exclusion criteria were as follows: a ROM limitation of the joints involved in ULNT1-STD and ULNT1-DIST, the presence of neurological disorders (e.g., multiple sclerosis), other suspected or confirmed peripheral nervous system disorders (e.g., diabetic neuropathy or cervical radiculopathy), severe conditions implicating physical contraindications for joint mobilization (e.g., infection, malignancies, or fractures), and surgeries that could potentially alter nerve biomechanics (e.g., nerve repair or CTS surgery).

The sample size was calculated using the statistical software Epidat 4.2 (Consellería de Sanidade, Xunta de Galicia, Spain; Pan American Health Organization (PAHO-WHO); Universidad CES, Colombia, 2016). A 95% confidence interval was established. The calculation was performed for a sensitivity and specificity of 80%, with an estimated prevalence of 54% [29] and an absolute error of 10%. The final sample size was determined to be 134 subjects.

### 2.3. Reference Test

Nerve conduction studies were conducted by a neurophysiologist following the AANEM (American Association of Neuromuscular & Electrodiagnostic Medicine) practice guidelines for CTS studies [5,60,61], using standard electrodiagnostic equipment.

Median nerve sensory NCSs were performed antidromically with the stimulation at the wrist 14 cm proximal to the recording electrode on the index finger, and included the assessment of sensory latency, conduction velocity, and sensory nerve action potential. Median nerve motor distal latency and compound muscle action potential were obtained by recording over the abductor pollicis brevis through orthodromic stimulation at the wrist (8 cm). If the NCS results were normal, conduction values of the median nerve were compared to the ulnar nerve in the same extremity; additionally, if considered necessary, other tests such as comparison short segment studies were performed [5,60,61]. The reference values reported by the AANEM’s Normative Data Task Force were considered for CTS diagnosis [62]. Hand temperature was monitored and maintained at least at 32° throughout the process.

### 2.4. Index Test

**Figure 1:**
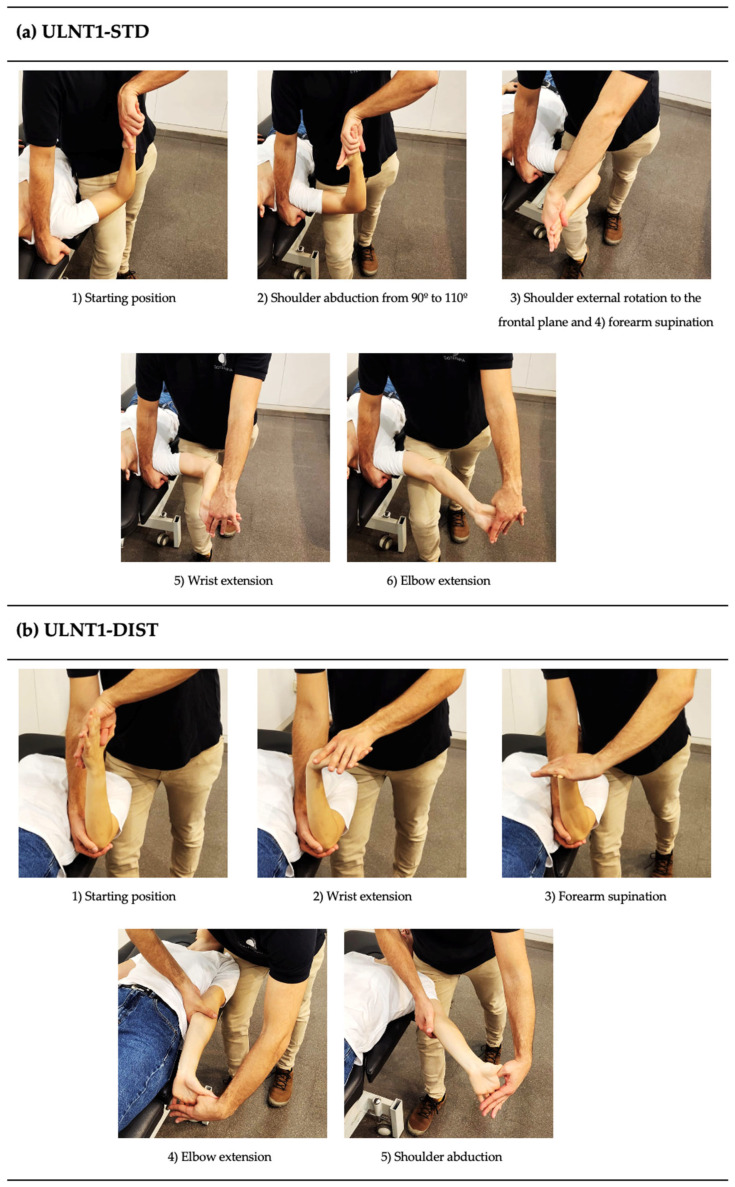
Joint movements and their respective order for (**a**) ULNT1-STD and (**b**) ULNT1-DIST. The tests were performed until symptom onset. Note that in both sequences, the scapula was stabilized in a neutral position to prevent elevation during the tests. ULNT1-STD = standard sequence of the upper limb neurodynamic test 1. ULNT1-DIST = distal-to-proximal sequence of the upper limb neurodynamic test 1.

Participants were positioned supine with the cervical spine in neutral position, the face in the horizontal plane, and the scapula in neutral position. ULNT1-STD and ULNT1-DIST were performed until the patient verbally reported the first appearance of a sensory response (P1). The movements and their respective order for each sequence are described in Figure 1. During the tests, the examiner stabilized the scapula above the participant’s shoulder to prevent any elevation. Particular care was taken to avoid applying scapular depression at any point in the sequence.

Once P1 was reached, an SD maneuver was performed by moving a body part away from the area of the response to modify neural tension [12,18,19,20,21,31,32,33,34,35,36,37,38]. If symptoms appeared in the elbow, forearm, wrist, or hand, contralateral cervical side flexion was used as SD for ULNT1-STD, while shoulder abduction release or scapular depression was the movement applied for ULNT1-DIST. If patients referred symptoms in the neck, shoulder, or arm, wrist dorsiflexion was released in both tests. If symptoms changed with SD, the test response was classified as “neurodynamic” (i.e., the neural tissue was implicated in symptoms); otherwise, it was considered “musculoskeletal” (i.e., symptoms were originated by non-neural tissues).

### 2.5. Definition of a Positive Index Test

Different criteria to determine a positive test were explored. In all criteria the symptomatic side must present a neurodynamic response (positive SD) in order to confirm neural involvement.

#### 2.5.1. Criterion 1—Symptom Reproduction

The tests were considered positive when a patient’s clinical symptoms were reproduced at least partially and these symptoms changed with SD. If the test evoked any other symptom, it was classified as negative (e.g., the patient’s usual symptoms are pain at the wrist and numbness in the hand, while the test produced a tingling response which the patient did not recognize as part of the usual symptoms) [20,21,51,63].

#### 2.5.2. Criterion 2—Symptom Reproduction or Range of Motion Asymmetry

The tests were considered positive when a patient’s clinical symptoms were reproduced at least partially (criterion 1). In case a patient’s symptoms were not reproduced, the test was considered positive if the symptomatic side presented ≤ 15° of the final ROM compared to the asymptomatic side [64]; or the test ended in an earlier stage in the symptomatic side (e.g., ULNT1-DIST ended during elbow extension in the symptomatic side, while the asymptomatic side reached the shoulder abduction stage). Positive SD in the symptomatic side was required in all cases.

#### 2.5.3. Criterion 3—Symptom Reproduction or Sensory Response Asymmetry

The tests were considered positive when patient’s clinical symptoms were reproduced at least partially (criterion 1). In case a patient’s symptoms were not reproduced, sensory response asymmetry was assessed in both the nature and the location; therefore the test was considered positive 1) if the participant reported a response of a neurogenic-like nature (tingling, pins and needles, numbness, burning, or electric shock) only in the symptomatic side; or 2) if the response was located in the median nerve area (wrist, palm, or first three fingers) only in the symptomatic side (e.g., the patient reported a sensation of “tension” in the thumb at the end of the test in the symptomatic side, while in the contralateral side, the patient felt the same sensation of “tension” at the elbow level). Positive SD in the symptomatic side was required.

Regarding this criterion, previous authors suggested analyzing symptom nature and location to detect abnormal responses to an NDT that could implicate an increase in nerve mechanosensitivity [18,42,63]. Supporting this, CTS patients showed a higher prevalence of distal responses during ULNT1 compared to non-CTS [51]. However, sensory responses located distally and with neurogenic-like nature are also reported in healthy subjects [38,42]; therefore, we decided to consider neurogenic-like responses and distally located responses abnormal only in comparison with the non-symptomatic side.

#### 2.5.4. Criterion 4—Symptom Reproduction or Range of Motion Asymmetry or Sensory Response Asymmetry

**Figure 2:**
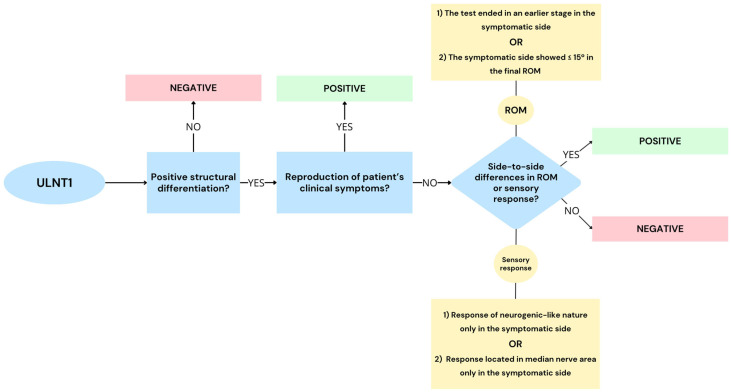
Decisional tree to determine a positive ULNT1 for carpal tunnel syndrome following criterion 4. ULNT1 = upper limb neurodynamic test 1. ROM = range of motion.

This criterion represents the combination of criteria 1, 2, and 3 (Figure 2). The test was considered positive if it reproduced at least part of the patient’s clinical symptoms. If a patient’s symptoms were not directly reproduced, the test was considered positive if asymmetry in ROM or sensory responses were found, according to criteria 2 and 3. Positive SD in the symptomatic side was required.

### 2.6. Range of Motion Measurement

The ROM of the final moved joint (elbow or shoulder) was recorded at the end of each ULNT1. Measurements were taken by the second examiner using a standard 360° two-arm goniometer. For elbow extension, the goniometer’s axis was positioned at the medial epicondyle of the humerus, with the stationary arm pointing toward the acromion and the moving arm aligned with the ulnar styloid [38,65]. Glenohumeral abduction angle was measured by placing the goniometer axis at the acromion, with the stationary arm parallel to the sternum and the moving arm aligned with the medial epicondyle of the humerus [66,67].

### 2.7. Data Collection

Before the assessments, patient history data, including age, sex, weight, height, dominant hand, and the characteristics and duration of symptoms, were collected. The first examiner, blinded to the NCS results and to the patient’s clinical history, performed both sequences of ULNT1, while the second examiner measured the final ROM of the last joint moved during the test using a goniometer. A 20–30 min interval was allowed between the NCS and the first test [20,27,29], and at least 10 min were left between the two tests [38,41]. After each ULNT1, patients marked the location of their symptoms on a body diagram and described their nature (tension, pain, burning, tingling, pins and needles, numbness, or other). The order in which the ULNT1 sequences were performed and limbs were tested was determined randomly by tossing a coin. The assessments were performed in a warm room maintained at a constant temperature of 21 °C. At the end of the procedure, the results of both ULNT1 sequences were compared to the NCS results.

### 2.8. Statistical Analysis

Descriptive statistics were calculated for demographic variables and clinical characteristics.

The ULNT1-STD and ULNT1-DIST intra-rater reliability for ROM measurement was investigated in 10 patients (age: 47.00 ± 14.31 years; 6 females and 4 males, affected side: 5 left and 5 right). The same test was performed twice with a ten-minute rest by the first examiner. The final ROM was recorded by the second examiner.

Two-way mixed effect model intraclass correlation coefficient “absolute agreement” was used to calculate the intra-rater reliability for both tests [68]. The standard error of measurement (SEM) and the minimal detectable change (MDC) were obtained as follows: SEM = standard deviation × $\sqrt{\left(1-ICC\right)}$; MDC = SEM × 1.96 × $\sqrt{2\;}$.

To assess diagnostic accuracy, sensitivity, specificity, positive predictive value (PPV), and negative predictive value (NPV) were calculated with 95% confidence intervals. The ULNT1 results with CTS diagnoses were compared with a two-by-two contingency table, and likelihood ratios (LRs) were calculated. The LRs were calculated as follows: +LR = sensitivity/(1 − specificity); −LR = (1 − sensitivity)/specificity [69,70].

Post-test probabilities were calculated using the LR with the following formulae: post-test odds = pre-test odds × likelihood ratio; odds = probability/(1 − probability); probability = odds/(1 + odds) [70]. Fagan’s nomograms were used to visually represent the change in post-test probability based on positive or negative ULNT1 results [70,71]. Data were analyzed using Microsoft Excel version 16.91 (Microsoft Corporation) and SPSS version 29.0 (IBM Corp., Armonk, NY, USA).

## 3. Results

### 3.1. Sample

**Figure 3:**
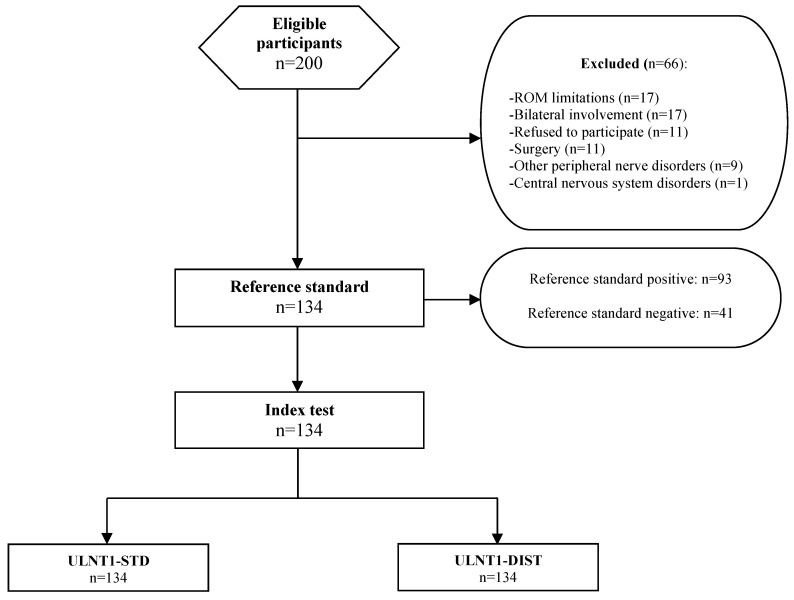
Flow chart of the study. ROM = range of motion. ULNT1-STD = standard sequence of the upper limb neurodynamic test 1. ULNT1-DIST = distal-to-proximal sequence of the upper limb neurodynamic test 1.

Two-hundred patients were initially evaluated for potential eligibility. Sixty-six patients were excluded. The final sample consisted of 134 consecutive patients (see Figure 3). The characteristics of the included participants are described in Table 1.

### 3.2. Intra-Rater Reliability

The intra-rater reliability for ROM measurements at the end of the test was assessed in both ULNT1 sequences for the symptomatic and the non-symptomatic side. For the ULNT1-STD sequence, the symptomatic side had an ICC of 0.959, with an SEM of 2.478° and an MDC of 6.868°. On the non-symptomatic side, the ICC was 0.851, the SEM 2.986°, and the MDC 8.277°. For the ULNT1-DIST sequence, the symptomatic side showed an ICC of 0.925, with an SEM of 4.464° and an MDC of 12.374°, while the non-symptomatic side presented an ICC of 0.980, an SEM of 2.580°, and an MDC of 7.152°.

### 3.3. Standard Sequence of the Upper Limb Neurodynamic Test 1

**Figure 4:**
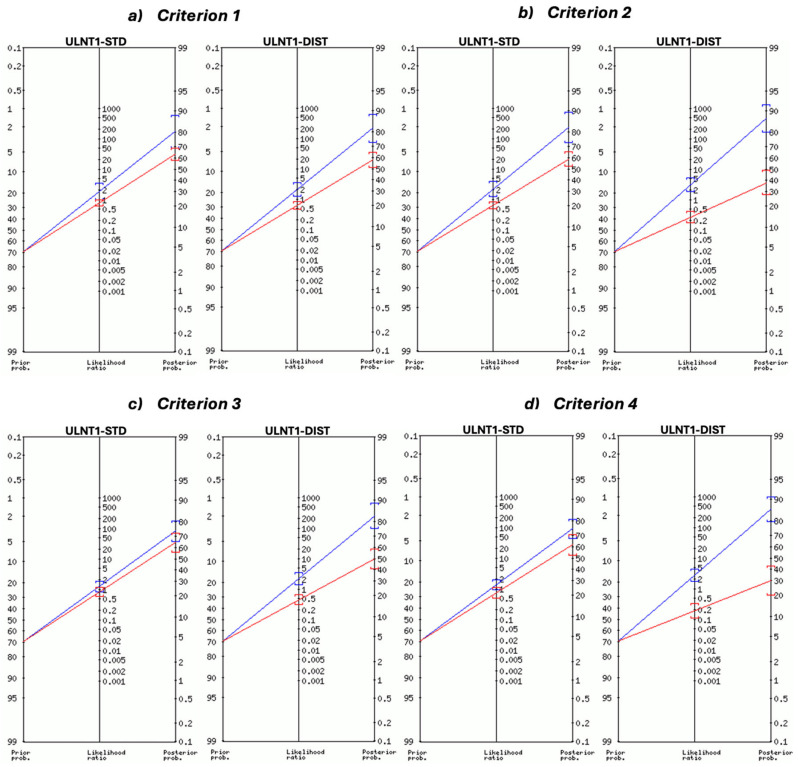
Fagan’s nomogram to graphically show the change from the pre-test probability (69%) using ULNT1-STD and ULNT1-DIST with (**a**) criterion 1—symptom reproduction; (**b**) criterion 2—symptom reproduction OR ROM asymmetry; (**c**) criterion 3—symptom reproduction OR response asymmetry; (**d**) criterion 4—symptom reproduction OR ROM OR response asymmetry. Positive SD in the affected limb was required in all criteria. Blue lines indicate the positive likelihood ratio with the corresponding post-test probability. Red lines indicate the negative likelihood ratio with the corresponding post-test probability.

The diagnostic accuracy of ULNT1-STD was evaluated using four different criteria (Table 2 and Table 3 and Figure 4). When considering symptom reproduction alone (criterion 1), sensitivity was relatively low (0.398 [95% CI: 0.299–0.505]), though specificity was higher at 0.780 (95% CI: 0.624–0.894). +LR for this criterion was 1.81 (95% CI: 0.97–3.40), and −LR was 0.77 (95% CI: 0.61–0.97). Criterion 2 resulted in an improvement in sensitivity to 0.516 (95% CI: 0.410–0.621), while specificity decreased slightly to 0.756 (95% CI: 0.597–0.876). +LR increased to 2.12 (95% CI: 1.19–3.76), and −LR decreased to 0.64 (95% CI: 0.49–0.84). With criterion 3, sensitivity improved slightly to 0.548 (95% CI: 0.442–0.652), but specificity declined to 0.561 (95% CI: 0.397–0.715). This resulted in a +LR of 1.25 (95% CI: 0.84–1.85) and a −LR of 0.81 (95% CI: 0.57–1.14), showing no significant diagnostic advantage. Finally, when using criterion 4, sensitivity increased to 0.613 (95% CI: 0.506–0.712), the highest among all criteria for ULNT1-STD, with a specificity of 0.537 (95% CI: 0.374–0.693). This resulted in a +LR of 1.32 (95% CI: 0.92 –1.91) and a −LR of 0.72 (95% CI: 0.49–1.91).

### 3.4. Distal-to-Proximal Sequence of the Upper Limb Neurodynamic Test 1

The results obtained for ULNT1-DIST are illustrated in Table 2 and Table 3 and Figure 4.

ULNT1-DIST showed overall higher diagnostic values compared to ULNT-STD. When considering criterion 1, sensitivity was 0.548 (95% CI: 0.442–0.652) and specificity 0.732 (95% CI: 0.571–0.858), with a +LR of 2.04 (95% CI: 1.19–3.50) and a −LR of 0.62 (95% CI: 0.46–0.83). Using criterion 2, sensitivity showed an increase to 0.806 (95% CI: 0.711–0.881) with no associated loss in specificity. This was reflected in a higher +LR of 3.01 (95% CI: 1.80–5.03) and a lower −LR of 0.26 (95% CI: 0.17–0.42). For criterion 3, sensitivity was 0.699 (95% CI: 0.595–0.790) and specificity 0.683 (95% CI: 0.519–0.819). +LR for criterion 3 was 2.20 (95% CI: 1.38–3.52), and −LR was 0.44 (95% CI: 0.30–0.64). Finally, ULNT1-DIST with criterion 4 achieved the highest sensitivity of 0.871 (95% CI: 0.785–0.931), with a specificity of 0.683 (95% CI: 0.519–0.819). Despite the slight decrease in specificity with respect to criteria 1 and 2, a +LR of 2.75 (95% CI: 1.74–4.33) and a −LR of 0.19 (95% CI: 0.11–0.33) suggested an overall highest diagnostic accuracy for criterion 4.

## 4. Discussion

This study aimed to evaluate the diagnostic performance of ULNT1-DIST compared to ULNT1-STD in detecting CTS. We also explored the added diagnostic value of side-to-side difference in sensory response and ROM. Our findings suggest that ULNT1-DIST, which started distally at the wrist, offers better diagnostic accuracy, with higher +LRs and lower −LRs, than ULNT1-STD.

Our results showed that when a patient’s clinical symptoms are not reproduced directly by ULNT1-DIST, incorporating asymmetry criteria for ROM and sensory response (criterion 4) can substantially improve the test sensitivity with only a slight decrease in its specificity, with a +LR of 2.75 (1.74–4.33) and a −LR of 0.19 (0.11–0.33), leading to a post-test probability of 86% with a positive test and a 30% if the test is negative. According to this, ULNT1-DIST with criterion 4 may represent a suitable test to add in the clinical evaluation process of patients with suspected unilateral CTS.

In contrast, when the symptom reproduction-only criterion was used (criterion 1), ULNT1-DIST led to a +LR of 2.04 (1.19–3.50) and a −LR of 0.62 (0.46–0.83), which, in terms of post-test probability, means that for a positive test, 82% may have CTS and for a negative test, 58% could present the disease. This more modest improvement from the pre-test probability (69%) indicates that relying exclusively on symptom reproduction offers less diagnostic value compared to using asymmetry criteria in our sample. This aligns with previous authors suggesting that the interpretation of an NDT should not only be focused on symptom reproduction but also include the assessment of other variables such as ROM and response characteristics [12,18,42,63].

The diagnostic accuracy of ULNT1 with neurodynamic sequencing for CTS detection has been previously investigated [51]. A sensitivity of 65.7% and a specificity of 95.7% were reported considering ULNT1 positive with symptom reproduction and their modification with SD (criterion 1 in our study) [51]. The discrepancy between the results of previous research and the present study may be explained by differences in the participants and in CTS diagnostic criteria. Bueno-Gracia et al. [51] used an absolute cutoff of 40 m/s for median nerve conduction velocity, whereas we included comparative NCS, which could have detected milder cases classified as negative in the mentioned study [72]. Moreover, in their study, participants with bilateral symptoms were included [51]. Previous research suggests that patients with bilateral CTS may be subjected to worse functional outcomes and a higher severity of symptoms [73]. The inclusion of participants with bilateral symptoms and the less sensitive reference standard used may have resulted in recruiting and diagnosing with CTS participants who were clinically more affected [73] and more likely to present a heightened nerve mechanosensitivity. Therefore, despite using the same ULNT1 sequence, the reproduction of neural-related symptoms in participants classified as positive for CTS could have been easier than in our study, explaining the higher diagnostic values found with respect to our study.

Despite the difference found in the diagnostics values, both studies support the idea that starting the neurodynamic sequence at the wrist may enhance diagnostic accuracy by stressing the median nerve more directly at its site of entrapment.

According to our findings, ULNT1-STD seems to be a clinically less useful test for CTS diagnosis regardless of the criteria used. Criterion 2, which included symptom reproduction or ROM asymmetries, showed the highest values among all ULNT1-STD criteria, with a +LR of 2.12 (1.19–3.76) and a −LR of 0.64 (0.49–0.84); nonetheless, it still implicated only minor changes in post-test probability. ULNT1-STD in our study yielded to a substantially lower sensitivity, but a higher specificity compared to many previous investigations [27,29,30]. This might be due to the diagnostic criteria adopted in these studies [27,29,30], which are more inclined to classify as positive many healthy subjects [18,38,42]. Another study defined a positive ULNT1 when the test reproduced a patient’s symptoms, and these symptoms were modified with structural differentiation (criterion 1 of our study) [20]. This last research obtained a sensitivity of 0.58 and a specificity of 0.84 [20], which is a closer result to the one found in our study.

An important aspect of our study was the incorporation of asymmetry as a diagnostic criterion, comparing the symptomatic to the asymptomatic side. This approach is supported by prior suggestions in the literature, which propose that differences in ROM or sensory response between limbs may indicate heightened nerve mechanosensitivity [18,26,42,63].

Noticeably, our results showed that this focus on asymmetry in ROM and sensory response improved the diagnostic performance for ULNT1-DIST but not ULNT1-STD.

It is possible that first moving the joints closest to the affected area not only facilitates symptom reproduction in less irritable cases but also helps to highlight subtle differences in nerve mechanosensitivity, such as reduced ROM or localized symptoms, which may be overlooked in the standard sequence.

Despite the fact that neurodynamic sequencing has been shown to produce different responses to neurodynamic testing in healthy subjects [74,75], its mechanism of action is still unclear. A suggested underlying mechanism is that a specific nerve segment may be subjected to a higher tension and compression for a longer time period and differences in the final position at the end of the test may contribute to an increase in segmental nerve mechanical stress with neurodynamic sequencing [48,50,51].

While the results for ULNT1-DIST incorporating asymmetry seem to be promising, it is important to emphasize that NDTs are not definitive diagnostic tools for CTS. Since during an NDT, tension progressively spreads along the neural tract, different nerve portions are stressed [55,76,77,78]. Consequently, NDTs appear to be better designed to assess altered mechanosensitivity rather than diagnosing a specific nerve pathology. Although determined movement sequences may enhance neural load in specific areas, they should be used as part of a comprehensive diagnostic process.

### Limitations

Several limitations should be addressed.

First, CTS frequently presents bilaterally, with approximately 60% of patients experiencing symptoms in both hands [79,80]. Our criteria for side-to-side comparisons (criteria 2, 3, and 4) do not account for this bilateral presentation, limiting the diagnostic utility of these comparisons in such cases.

Additionally, participants were not generally assessed bilaterally with NCSs. According to the AANEM guidelines, when NCSs are abnormal in one limb and that limb is the only symptomatic limb, NCSs on the opposite hand are not recommended [61]. This raises the possibility that some subjects with subclinical nerve alterations on the asymptomatic side may have been included [81], which could potentially have reduced the accuracy of side-to-side comparisons, leading to an underestimation of the diagnostic performance of the tests. However, in clinical practice, provocation tests such as ULNT1 are often conducted before NCS, so the potential presence of subclinical alterations on the asymptomatic side in our study reflects a typical clinical scenario.

Moreover, patients were tested after the NCS. Despite that a time lapse of 20–30 min was left before performing the first ULNT1, it cannot be excluded that NCS may have altered the sensitivity of the median nerve and potentially biased the results of the tests.

Lastly, we adopted the 15° cutoff for interlimb differences in ROM proposed by Stalioraitis et al. [64] for both test sequences. This cutoff was specifically proposed for elbow extension in the standard ULNT1 sequence. However, we extended it to ULNT1-DIST to simplify the interpretation and comparisons between the two sequences. Future research should investigate the diagnostic accuracy of different ROM cutoffs for interlimb comparisons in both sequences.

## 5. Conclusions

Our findings support the hypothesis that ULNT1-DIST offers better diagnostic accuracy for CTS compared to the ULNT1-STD sequence, especially including the assessment of ROM and sensory response asymmetry. However, the diagnostic utility of side-to-side comparisons does not account for patients with a bilateral presentation of symptoms, limiting their application in clinical practice. Despite the fact that determined movement sequences may focus neural load in specific areas, NDTs should be used as part of a comprehensive diagnostic process. 

## Figures and Tables

**Table 1:** Participants’ characteristics. Continuous data are represented as mean ± standard deviation; categorical data are represented as percentages.

Total	n = 134
Females	77.6%
Age (Years)	55.47 ± 13.49
Hand dominance	95.5% right
Height (m)	1.63 ± 0.85
Weight (kg)	73.40 ± 17.08
BMI	27.53 ± 5.59
Symptomatic side	70.1% right
Symptom duration	
<1 month	0 (0%)
1–3 months	7 (5%)
>3 months	127 (95%)
Type of principal symptoms	
Numbness	70.9%
Paresthesia	64.9%
Pain	37.3%
Electric shock	5.2%
Muscle weakness	3.7%
Night pain	82.1%
CTS diagnosis	69.4%

BMI = body mass index. CTS = carpal tunnel syndrome.

**Table 2:** Two-by-two contingency tables for ULNT1 sequences and reference test.

		Positive CTS	Negative CTS	
**ULNT1-STD**				
				Total
Criterion 1	Positive	37	9	46
	Negative	56	32	88
	Total	93	41	134
Criterion 2	Positive	48	10	58
	Negative	45	31	76
	Total	93	41	134
Criterion 3	Positive	51	18	69
	Negative	42	23	65
	Total	93	41	134
Criterion 4	Positive	57	19	76
	Negative	36	22	58
	Total	93	41	134
**ULNT1-DIST**				
				Total
Criterion 1	Positive	51	11	62
	Negative	42	30	72
	Total	93	41	134
Criterion 2	Positive	75	11	86
	Negative	18	30	48
	Total	93	41	134
Criterion 3	Positive	65	13	78
	Negative	28	28	56
	Total	93	41	134
Criterion 4	Positive	81	13	94
	Negative	12	28	40
	Total	93	41	134

ULNT1-STD = standard sequence of the upper limb neurodynamic test 1. ULNT1-DIST = distal-to proximal sequence of the upper limb neurodynamic test. CTS = carpal tunnel syndrome.

**Table 3:** Diagnostic accuracy of ULNT1-STD and ULNT1-DIST for carpal tunnel syndrome detection. All values are shown with their respective 95% confidence intervals. The post-test probability values are based on a pre-test probability of 69%.

	Sensitivity	Specificity	PPV	NPV	+LR	−LR	PPPT	PPNT
ULNT1-STD *								
1—Symptom reproduction	0.398 (0.299–0.505)	0.780 (0.624–0.894)	0.804 (0.687–0.885)	0.364 (0.312–0.419)	1.81 (0.97–3.40)	0.77 (0.61–0.97)	80% (69–89)	64% (58–69)
2—Symptoms OR ROM asymmetry	0.516 (0.410–0.621)	0.756 (0.597–0.876)	0.828 (0.730–0.895)	0.408 (0.344–0.475)	2.12 (1.19–3.76)	0.64 (0.49–0.84)	83% (73–90)	59% (53–66)
3—Symptoms OR Response asymmetry	0.548 (0.442–0.652)	0.561 (0.397–0.715)	0.739 (0.657–0.807)	0.354 (0.278–0.438)	1.25 (0.84–1.85)	0.81 (0.57–1.14)	74% (66–81)	65% (56–72)
4—Symptoms OR ROM OR Response asymmetry	0.613 (0.506–0.712)	0.537 (0.374–0.693)	0.750 (0.675–0.812)	0.379 (0.294–0.472)	1.32 (0.92–1.91)	0.72 (0.49–1.06)	75% (68–81)	62% (53–71)
ULNT1-DIST *								
1—Symptom reproduction	0.548 (0.442–0.652)	0.732 (0.571–0.858)	0.823 (0.730–0.888)	0.417 (0.348–0.489)	2.04 (1.19–3.50)	0.62 (0.46–0.83)	82% (73–89)	58% (51–65)
2—Symptoms OR ROM asymmetry	0.806 (0.711–0.881)	0.732 (0.571–0.858)	0.872 (0.803–0.919)	0.625 (0.514–0.724)	3.01 (1.80–5.03)	0.26 (0.17–0.42)	87% (80–92)	37% (28–49)
3—Symptoms OR Response asymmetry	0.699 (0.595–0.790)	0.683 (0.519–0.819)	0.833 (0.758–0.888)	0.500 (0.408–0.592)	2.20 (1.38–3.52)	0.44 (0.30–0.64)	83% (76–89)	50% (40–59)
4—Symptoms OR ROM OR Response asymmetry	0.871 (0.785–0.931)	0.683 (0.519–0.819)	0.862 (0.798–0.908)	0.700 (0.569–0.805)	2.75 (1.74–4.33)	0.19 (0.11–0.33)	86% (80–91)	30% (20–43)

* In all criteria, the test must show a positive structural differentiation in the symptomatic side. ULNT1-STD = standard sequence of the upper limb neurodynamic test 1. ULNT1-DIST = distal-to-proximal sequence of the upper limb neurodynamic test 1. ROM = range of motion. PPV = positive predictive value. NPV = negative predictive value. +LR = positive likelihood ratio. −LR = negative likelihood ratio. PPPT = post-test probability for a positive test. PPNT = post-test probability for a negative test.

## Data Availability

The datasets presented in this article are not readily available because the data are part of an ongoing doctoral thesis. Requests to access the datasets should be directed to the corresponding author.
